# Managing information in eye care programmes: the health systems perspective

**Published:** 2010-12

**Authors:** Hannah Faal, Colin Cook, RD Thulasiraj

**Affiliations:** Programme Development Adviser: Health Systems, Sightsavers, 21 Nii Nortei Ababio Road, PO Box KIA 18190, Airport, Accra, Ghana.; Professor of Ophthalmology, University of Cape Town; CBM Eye Medical Advisor.; Executive Director, LAICO, Aravind Eye Care System; President, VISION 2020: The Right to Sight: India, Lions Aravind Institute of Community Ophthalmology, Aravind Eye Care System, Annanagar, Madurai 625 020, Tamil Nadu, India.

**Figure:**
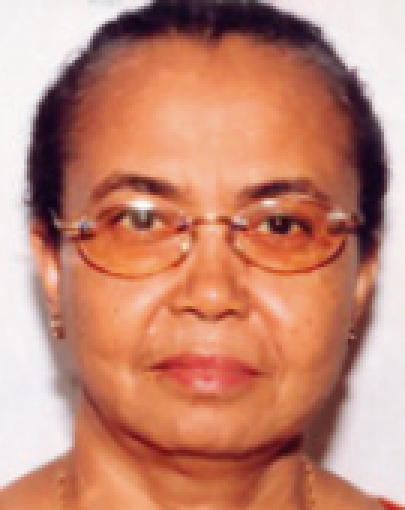


**Figure:**
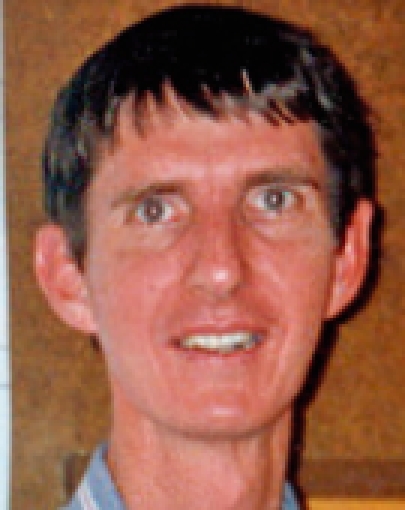


**Figure:**
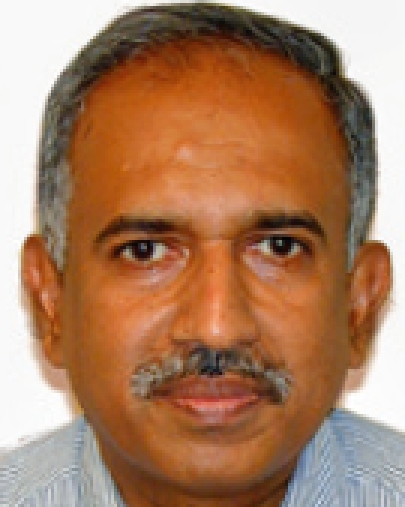


## What is the health systems perspective?

As eye care workers, we don't treat eyes, we treat people. Similarly, eye care does not take place in a vacuum; it is part of the wider health care system.

It is possible to forget this ‘bigger picture’ when our work demands such a lot of us. However, as we think about it, we will be surprised how much the work we do depends on the wider health system and, in turn, how much our eye care system can contribute to the wider health system.

The World Health Organization emphasises the need to have the health systems perspective or framework in mind when we plan and deliver eye care services.

This is not as complicated as it may sound! It means spending a little time thinking about each of the different but related elements that make up the building blocks of the health system.

1. **Service delivery:** the actions we take to improve the health of our patients
2. **Human resources:** the people who deliver eye care
3. **Consumables and technology:** the tools we need in order to do our work
4. **Financing:** how the eye care is funded
5. **Leadership and governance:** how we manage our work, are accountable, and how our work is regulated
6. **Health information:** the information we need to manage our work.

We can think about each of these within our eye programme. But more importantly, the health systems perspective encourages us to think about how our eye programme interacts - both positively and negatively - with nearby, parallel health services and the wider health system such as the local hospital or national health system.

**Figure:**
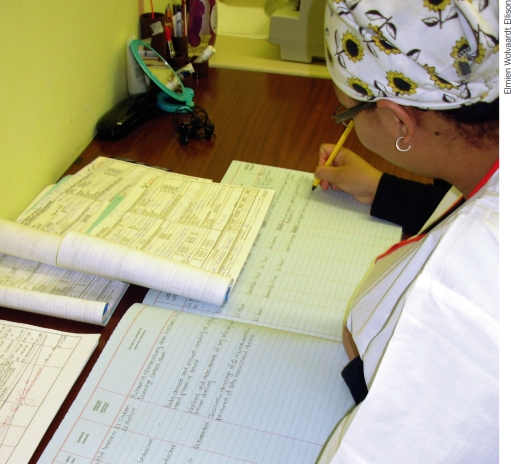
We need accurate information to plan and monitor eye services. SOUTH AFRICA

Elmien Wolvaardt Ellison

## Health management information systems (HMIS)

Health information is one of the six components of a health care system. We need information about each of the components in order to plan and monitor - with the ultimate aim of providing a better service and eliminating avoidable blindness.

A health management information system (HMIS), if well designed, can help us to manage all the information needed by (and generated by) the eye care programme - whether at local, district, or national level. Whether we are planning a new HMIS or evaluating an existing HMIS, the health systems framework can be a useful thinking tool.

It can help us to determine what questions the HMIS should be able to answer (which in turn determines how we design it) and plan the implementation of our HMIS.

Central to both of these is an awareness of how our planned HMIS will integrate with any others in existence; or, where there are no others, how ours can be extended to support other health services.

## What questions should your HMIS be able to answer?

1. **Service delivery**
  In order to know how well we are doing, we need information on how many people we are reaching (the quantity). Simple counts (or tallies) of the number of patients seen, screened, referred, and treated over the last day, month, or year will give us some idea of how we are doing. Information about the quality of our services is equally important. Table 1 gives some examples of the information we can collect to monitor the quantity and quality of our services. The purpose of collecting this information is either to reinforce that we are on the right track and/or to identify areas that need improvement or change.
  It is vital that this information is shared with those who are responsible for the work behind the numbers. This is both to acknowledge work well done and to plan ways of improving our work where necessary. The information collected should also be used by us and not just be passed on to others. The authors recommend holding periodic review meetings to analyse the information, identify problems or constraints, and decide on the steps we must take to do better.
  At the level of the individual eye unit, we recommend that the person who collects the information should also analyse and present it. For example, the person who completes the daily register could be trained to analyse the data and present it at the monthly eye care team meeting. This will help them collect the information more carefully. They could look at how common different conditions are or the areas most patients come from. This will bring life to what could otherwise be a boring duty.
2. **Human resources**
  Your HMIS should try to capture the following:
  - Who is employed where, and to do what? In collecting information on human resources, you should look at all workers who contribute to the work, not just health workers. For example, you should consider records clerks, equipment technicians, and administrators in different districts.
  - How many patients are seen at different eye units and by different staff members?
  - Where are there long waiting times? You may improve the flow of patients by moving staff within a particular hospital, or by assigning staff to a clinic or hospital where there are more patients and longer waiting times and waiting lists.
3. **Consumables and technology**
  The HMIS may be able to help you keep track of stock levels and stock used (this will be covered in detail in Issue 76, December 2011), as well as what equipment is available and functioning (see Issue 73, September 2010).
4. **Financing**
  Your HMIS should capture information to produce financial reports which can be used to manage income generated, to manage budgets, and for reports to donors.
5. **Leadership and governance**
  A summary of the above information will be invaluable to make decisions about all aspects of the eye programme and help you when reporting to your manager, the hospital leadership, the ministry of health, or a donor. It is good to spend time thinking about your reporting requirements when you design the HMIS.

**Table 1:** Examples of information for monitoring the quantity and quality of eye care services provided

Eye care services	Information for monitoring quantity of work	Information for monitoring quality of work
**Trachoma**	Number of patients seen requiring medical treatment	Proportion of people needing services who are coming for treatment (are we meeting the need?)
	Number of trichiasis operations done	Number of repeat trichiasis operations done
**Refractive error**	Number of refractions done	Proportion of patients with 6/6 distance corrected vision
	Number of glasses prescribed or dispensed	Proportion of patients with J2 near corrected vision
**Cataract**	Number of cataract operations performed	Proportion of eyes achieving vision 6/18 or better at last postoperative visit
	Number of cataract operations performed on blind patients	Proportion of eyes failing to see 6/60 at last postoperative visit

**Figure:**
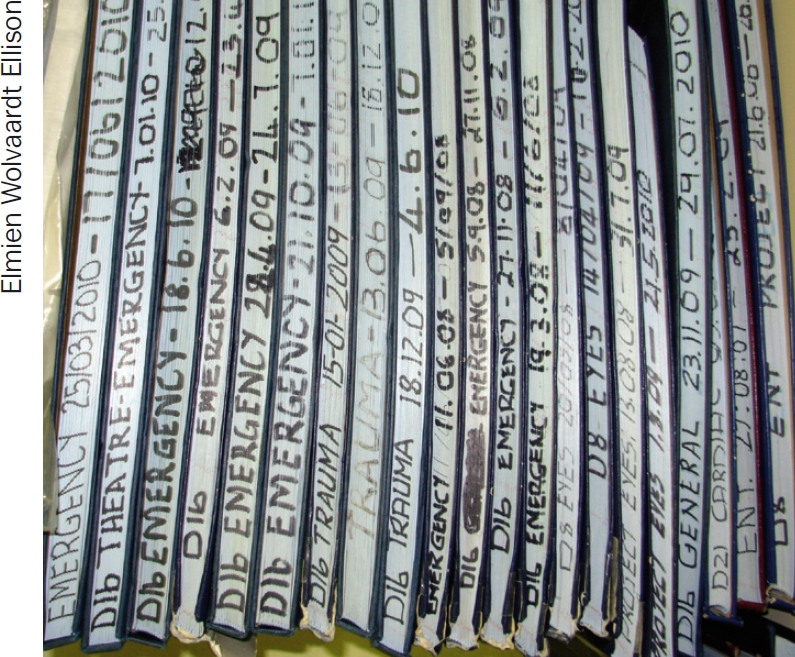
Do not destroy existing systems, but build on their strengths.

Elmien Wolvaardt Ellison

**Steps for developing or improving an HMIS**

(Adapted from *Developing health management information systems: a practical guide for developing countries* by WHO Western Pacific Region)

1. **Review the existing system.**
  - Make an inventory of the forms, log books and other tools used to record and summarise data at different levels.
  - Assess the quality of the data being collected using the existing forms/ formats at different levels.
  - Determine the problems encountered with the current system of data collection, including problems with timing and flow of information.
2. **Define the data needs of relevant units within the health system.**
  Define the different roles/functions of each level, for each of the major programmes; these will determine what information they need. For example, at the village level, information may be needed for case finding and service delivery; at the district level, information may be needed for monitoring and supervision. At the provincial level, information may be needed for programme planning and evaluation, and at the national level, for policy formulation.
3. **Determine the most appropriate and effective data flow.** Determine what data will be submitted to whom, how frequently, and in what form. Make a flow chart that shows the flow of information from the peripheral to the highest level.
4. **Design the data collection tools.**
  - Develop a first draft of the form, ensuring that it will produce all the data you need.
  - Get feedback from staff who will be using the form and make improvements where necessary.
5. **Develop the procedures and mechanisms for data processing.**
  - Assess the advantages and disadvantages of manually processing the data compared to using computers. Consider the cost, the availability of personnel (and their training, particularly at the lowest level), as well as the availability of technical support.
  - Pre-test any software and develop a training programme for staff.
6. **Develop and implement a training programme for data providers and data users.** This should include training of trainers, data providers, and computer operators and training of staff in the use of data generated by the system (at various levels).
7. **Pre-test and, if necessary, redesign the system for data collection, data flow, data processing and data use.**
8. **Monitor and evaluate the system once it is in use.**
9. **Develop effective data dissemination and feedback mechanisms.**
  Feed back to staff involved in the HMIS not only its outputs, but also who is using the information, what they are using and how.
10. **Enhance the HMIS.** Use the results of the monitoring and evaluation of the HMIS to continually improve it and decide on future expansion, where appropriate.

**Useful tips**

- Do not destroy existing systems; rather build on the strengths and learn from the weaknesses of what already exists.
- Consult with staff when you design the forms and/or computer system so that you are sure they understand them and know how to use them.
- Collect only the data which you are sure you will use.
- Ensure that the people collecting the data understand what it will be used for. Report the results back to them on a regular basis - this will help to keep staff motivated.
- The most effective data collection and reporting tools are simple and short.
- Data which is incomplete or incorrectly collected is worse than useless - it can give you false information.
- Not all data should be generated through the routine system of data collection. Data that are not frequently needed or are required only for certain subsets of the population can be generated through special studies and sample surveys.
- The development of the HMIS is always a work in progress. It is a dynamic endeavour where managers and workers strive for constant improvement.

