# Supplementary material for: Managing information in eye care programmes: the health systems perspective
Source: Community Eye Health. 2010 Dec;23(74):50–2. (PMC3033615)
Supplement: Related texts

# Brief report: Case study: redesigning Kenya's HMIS for eye care

**Published:** 2010-12

**Authors:** Wanjiku Mathenge

**Affiliations:** Regional Medical Advisor, Fred Hollows Foundation. Email: **ciku@email.com**

In Kenya, the Division of Ophthalmic Services is responsible for coordinating eye care services in the country. To do this, it relies on data which, until recently, had been collected manually, sent by post, and then recorded centrally. This process has been fraught with difficulties: data was often incomplete and there was a low response rate from eye units (39% in 2007).

There was a need to enhance the health management information system to improve data collection and present data to the Division of Ophthalmic Services in a clear and understandable format that would facilitate planning.

The aim of the redesign was to ensure that the system was simple to use and did not require advanced computer skills.

## Development of the HMIS

Microsoft Access was chosen as the operating platform. The new HMIS system was designed to allow data to be keyed into the computer at the health facility and relayed by mobile phone modem to the centralised database hosted by a server at the Division of Ophthalmic Services. The same data would be synchronised and integrated with the server at the Ministry of Health.

The Fred Hollows Foundation provided funding to:

- develop the software with feedback from users
- provide computers for eye units at provincial hospitals
- provide modems from a local mobile phone network.

## Features

- Eye units enter routine information they would have collected anyway.
- Hospitals or clinics can access their own data and use it to generate their own reports.
- The Division of Ophthalmic Services can use the system to produce reports based on facility, district, or province as well as disease category, treatment, or type of operation.
- Authorised users can develop data sets for children or women only, or for a specific disease.

## Progress

The system was piloted in three hospitals and has so far been implemented in nine hospitals in total. Once the seven provincial eye and three referral tertiary facilities in Nairobi are connected, it will be expanded to the 46 eye units at district level.

**Figure:** Staff being trained to use the HMIS. KENYA

Wanjiku Mathenge

## Outcomes

The system has enabled timely and useful information to be available, such as childhood morbidity and causes of blindness and low vision. The accuracy of reports has improved and the planning of eye services has been facilitated nationally and regionally.

## Challenges faced

The process has not been easy! Uptake has been very slow and several problems have been faced in the implementation as well as in the use of the system in the nine hospitals where the system is now installed. Dedicated use of modems for data entry only has reduced the use or airtime and brought down the cost to US $3 per month. Slow connection speeds have prevented use of the patient module but a dedicated server and line would eliminate this problem. The use of different HMIS software programmes throughout Kenya remains a problem.

## Lesson learnt: overcoming resistance

Eye care workers initially showed resistance to the amount of extra work that would be needed. The fact that they could access the information they entered and use the system to create their own reports made a big difference. Once they saw that the system could help them with planning and with filling out the newly introduced yearly performance reports, they embraced the idea and were happy to use the system.
